# Pirfenidone Inhibits Hypoxic Pulmonary Hypertension through the NADPH/ROS/p38 Pathway in Adventitial Fibroblasts in the Pulmonary Artery

**DOI:** 10.1155/2020/2604967

**Published:** 2020-06-11

**Authors:** Song Zhang, ZongXiu Yin, WeiDong Qin, XiaoLi Ma, Yao Zhang, EnXiu Liu, YanBiao Chu

**Affiliations:** ^1^Department of Pulmonary and Critical Care Medicine, Shandong Provincial Hospital Affiliated to Shandong First Medical University, Jinan, China; ^2^Department of Pulmonary and Critical Care Medicine, Jinan Central Hospital, Shandong First Medical University & Shandong Academy of Medical Sciences, Jinan, China; ^3^Department of Pulmonary and Critical Care Medicine, Jinan Central Hospital Affiliated to Shandong University, Jinan, China; ^4^Department of Critical Care Medicine, Qilu Hospital, Shandong University, Jinan, China; ^5^Central Laboratory, Jinan Central Hospital Affiliated to Shandong University, Jinan, China

## Abstract

Hypoxic pulmonary hypertension (HPH) is a devastating disease characterized by progressive vasoconstriction and vascular remodeling. Pirfenidone (PFD) inhibits the progression of HPH, though the molecular mechanisms remain unknown. This study is aimed at determining the role and mechanism of PFD in HPH in human pulmonary artery adventitial fibroblasts (HPAAFs), which were cultured under normal or hypoxic conditions. NOX4 and Rac1 were inhibited or overexpressed by shRNA or pcDNA3.1, respectively. Proliferation of HPAAFs was quantified by colorimetric 3-(4,5-dimethylthiazol-2-yl)-2,5-diphenyltetrazolium (MTT) assays to assess cellular metabolic activity, cell counts, and ethynyldeoxyuridine (EdU) assays to detect DNA synthesis. Migration of HPAAFs was assessed by a wound healing assay. The expression levels of smooth muscle alpha-actin (a-SMA) and procollagen I (COL1A1) were assessed by RT-PCR and western blot analysis. PFD suppressed hypoxia-induced proliferation and migration of HPAAFs. Compared with the hypoxic control group, PFD reduced the expression of a-SMA and procollagen I (COL1A1). PFD reduced hypoxia-induced phosphorylation of p38 through the NOX4/reactive oxygen species (ROS) signaling pathway. Moreover, Rac1 also decreased hypoxia-induced phosphorylation of p38, without any cross-interaction with NOX4. These findings demonstrate that PFD is a novel therapeutic agent to prevent cell proliferation, migration, and fibrosis, which might be useful in inhibiting vascular remodeling in patients with HPH.

## 1. Introduction

Hypoxic pulmonary hypertension (HPH) is a complicated multifactorial syndrome characterized by pulmonary vascular remodeling, and its treatment is largely palliative [1, 2]. The extensive remodeling of the pulmonary artery (PA) occurs in all three layers of the vascular wall, including the intimal endothelial cells, medial smooth muscle cells, and adventitial fibroblasts. Importantly, the vascular adventitia is the most heterogeneous portion, containing conduits for nutrient supply, such as the vasa vasorum, lymphatic vessels, and trophic nerves, as well as resident cells, such as fibroblasts, progenitor cells, and immune cells [3]. The adventitia plays a key regulatory function in the release, retrieval, integration, and storage of nutrients in the vascular wall [2, 4]. At present, the proliferation and differentiation of pulmonary artery adventitial fibroblasts (PAAFs) are thought to be a critical mechanism for initiating the development of HPH [5]. In response to chronic hypoxia, PAAFs first become activated and differentiate into myofibroblasts, then proliferate, ultimately stimulating the recruitment of inflammatory cells and the release of key regulators [6]. Previous studies have suggested that the proliferation and differentiation of PAAFs during chronic hypoxia are mainly dependent on signal transduction of the p38 pathway and its downstream mediator, hypoxia-inducible factor 1 (HIF-1) [7–9]. Low-dose fluvastatin significantly suppresses p38 activity in bovine PAAFs under hypoxic condition, thereby inhibiting their excessive proliferation [10].

Reactive oxygen species (ROS) are also important regulators of p38 signaling in various cell types, including PAAFs. Hypoxic conditions can lead to increased expression of multiple isoforms of nicotinamide adenine dinucleotide phosphate (NADPH) oxidases (NOX) in human PAAFs, the most crucial isoform being NOX4 [11]. NOX proteins are multi-subunit enzyme complexes including the small guanosine-5′-triphosphate (GTP) binding protein Rac1 [11]. When Rac1 is activated, Rac-GTP translocates to the membrane to interact and form a complex with NADPH. The intracellular ROS are mainly produced by this NOX4 complex [12, 13]; however, to date, no drugs that directly act on NOX4 or p38 have been used to treat HPH patients.

Pirfenidone (PFD) is a small molecule that is very soluble in alcohol and aqueous solutions and is able to move through cell membranes without requiring a receptor for transport [14]. Several studies have reported that PFD exerts an antifibrotic effect in animal tissues including the lung, heart, skin, liver, and kidney [15, 16]. Recently, experimental and clinical evidence suggests that PFD may safely slow or inhibit the progression of lung fibrosis, especially idiopathic pulmonary fibrosis [17, 18]. However, it remains unclear whether PFD exerts protective effects in HPH, and the precise mechanisms of action are unknown.

In this experiment, PAAFs were stimulated by hypoxia, then their proliferation, migration, and fibrosis were assessed to investigate the role and mechanism of PFD in HPH, which could identify a novel therapeutic target for the treatment of HPH.

## 2. Materials and Methods

### 2.1. Cell Culture

HPAAFs were purchased from ScienCell (CA, USA) and grown in fibroblast medium (ScienCell, CA, USA) consisting of 2% fetal bovine serum (FBS), 1% fibroblast growth supplement, and 1% penicillin/streptomycin. Cells were grown at 37°C in a 5% CO_2_ incubator and used between passages 3 and 8. The cells were exposed to hypoxic conditions inside a standard culture chamber (Whitley H35 Hypoxystation, Don Whitley Scientific, England), with an atmosphere of 5% O_2_, 5% CO_2_, and 90% N_2_. Pirfenidone (PFD) was added to cells at a concentration of 0.1mg/ml or 0.2mg/ml for 12 h or 24 h before hypoxia.

### 2.2. Gene Interference

HPAAFs were plated at a density of 10^5^ cells/well on a 24-well plate. The shRNA of NOX4 (5′-CCGGAACGAAGGGGTTAAACACCTCCTCGAGGAGGTGTTTAACCCCTTCGTTTTTTTG-3′) and Rac1 (5′-CCGGCAAACAGACGTGTTCTTAATTCTCGAGAATTAAGAACACGTCTGTTTGTTTTTG-3′) and pcDNA3.1 of NOX4 and Rac1 were synthesized and transfected into cells by using Lipofectamine® 3000 (Invitrogen, MO, USA), as previously described [19, 20]. After 48 h of transfection, the cells were treated with PFD and hypoxia was induced. Western blot analysis was used to confirm the interference efficacy.

### 2.3. MTT Assay

To assess cellular metabolic activity, the HPAAFs were seeded into 96-well plates at a density of 10^4^ cells/well. After stimulation, the MTT solution was added to each well for a 2 h incubation. Following removal of media, the formazan crystals were dissolved in 100 *μ*l of DMSO (Sigma-Aldrich, MO, USA). Cell viability was assessed by measuring the absorbance at 595 nm using a spectrophotometer (Thermo Scientific, MO, USA). The relative cell viability was calculated and compared with the absorbance of the untreated control group.

### 2.4. EdU Assay

To quantify cell proliferation, an EdU Kit (RiboBio, Guangzhou, China) was used according to the manufacturer's protocol. HPAAFs were seeded into 24-well plates with a glass slide in each well. Cells were serum-starved, then stimulated by hypoxia as described previously.

### 2.5. Cell Counting

HPAAFs were starved in serum-free media for 24 h, then stimulated by hypoxia. The cells were washed with PBS, and the number of cells was determined by counting in 10 random fields.

### 2.6. Wound Healing Assay

Wound healing culture inserts were used to analyze cell migration. According to the manufacturer's instructions, HPAAFs were plated at a concentration of 1 × 10^5^ cells/ml and incubated for 24 h, after which the culture inserts were removed. In order to assess migration of cells into the scratch area, images were taken every 6–12 h until closing of the scratch area could be confirmed visually under a microscope. Wound healing was analyzed using TScratch software. Each assay was repeated in triplicate.

### 2.7. Real-Time Quantitative Polymerase Chain Reaction (RT-PCR)

Total RNA was extracted from cells using Trizol (Invitrogen, Carlsbad, CA), and transcript levels were measured via SYBR Green-based real-time PCR using the SYBR Green Supermix (Bio-Rad Laboratories, CA, USA). Gene expression was normalized to GAPDH. Each sample was run in triplicate. The primer sequences used in this experiment were as follows: 5′-ACCGTATGCAGAAGGAAATCA-3′ (forward) and 5′-GCTAGAAACAGAGCAGGGAAGT-3′ (reverse) for a-SMA; 5′-GAGGGCCAAGACGAAGACATC-3′ (forward) and 5′-CAGATCACGTCATCGCACAAC-3′ (reverse) for procollagen I (COL1A1); and 5′-AGAAGGCTGGGGTCATTTG-3′ (forward) and 5′-AGGGGCCATCCACAGTCTTC-3′ (reverse) for GAPDH.

### 2.8. Western Blot Analysis

Cells were washed with ice-cold phosphate-buffered saline and lysed in radioimmunoprecipitation assay buffer (RIPA) (Thermo Scientific, MO, USA) with a Halt protease inhibitor and phosphatase inhibitor cocktail (Thermo Scientific, MO, USA). After being centrifuged, supernatants were collected and protein concentrations were determined by the bicinchoninic acid assay (BCA) (Thermo Scientific, MO, USA). Equal amounts of total protein were size-fractionated by 10% sodium dodecyl sulfate- (SDS-) polyacrylamide gel. After blocking with 5% silk milk, the blots were immunoblotted with corresponding antibodies. After washing, proteins were incubated with horseradish peroxidase- (HRP-) conjugated secondary antibodies, and protein bands were visualized by the use of a chemiluminescent HRP substrate (Millipore, MA, USA). NADPH expression was used to ensure equal protein loading.

### 2.9. Determination of Cellular ROS Levels

#### 2.9.1. DCFH Technique

2′,7′-Dichlorofluorescin diacetate (DCFH-DA) (DCF-DA, Molecular Probes, Eugene, OR, USA) can be used to detect cellular ROS generation as described previously [21]. Once it had been taken up by cellular esterases, 2′,7′-dichlorofluorescin diacetate (DCFH-DA) was cleaved by cellular esterases to 2′, 7′-dichlorofluorescin (DCFH), which was then oxidized to the fluorescent product 2′,7′dichlorofluorescein (DCF). Cells were then plated on 6-well cell culture plates under normoxic (21% O_2_, 5% CO_2_) or hypoxic conditions (5% O_2_, 5% CO_2_), and another additional for cells washed and incubated with DCFH-DA (30 *μ*m) in serum-free culture medium. Being washed three times in serum-free culture medium, cells were lysed in liquid nitrogen and centrifuged. A spectrofluorometer was applied to detect the fluorescence of the supernatant at 535 nm with an excitation wavelength of 485 nm.

#### 2.9.2. Amplex Red Technique

Amplex Red Hydrogen Peroxide/Peroxidase Assay (Invitrogen, Molecular Probes, Eugene, OR) can measure cellular reactive oxygen species (ROS) generation as previously described [12]. The Amplex Red reagent (10-acetyl-3,7-dihydroxyphenoxazine) can react with H_2_O_2_ and generate a fluorescent product due to horseradish peroxidase. Cells were washed once in serum-free medium and lysed in liquid nitrogen after culturing in normoxic or hypoxic conditions. 50 *μ*l of the Amplex Red Reagent/HRP was added and kept from light in the room temperature for 30 min. The spectrofluorometer was used to measure the fluorescence at 595 nm with an excitation wavelength of 530 nm.

### 2.10. Statistical Analysis

SPSS 13.0 was used for statistical analysis. With normally distributed data, unpaired *t*-tests were employed for comparison of 2 groups. One-way analysis of variance (ANOVA) was used to compare multiple groups. All data were represented as means ± SEM (standard error of the mean), and a *P* value of <0.05 was considered statistically significant.

## 3. Results

### 3.1. Pirfenidone Inhibited Hypoxia-Induced PAAF Proliferation

**Figure 1:**
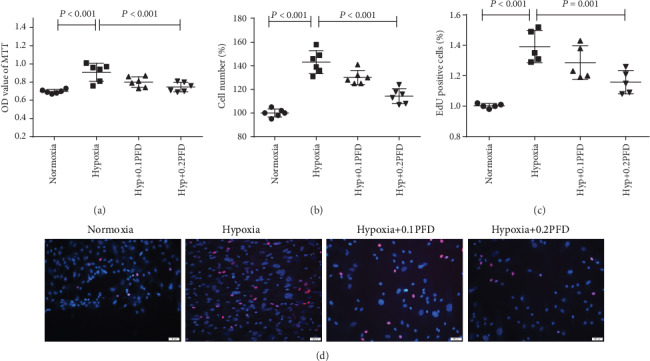
PFD reduced the proliferation of HPAAFs in hypoxia. HPAAFs were stimulated by PFD before hypoxia, and then, cell proliferation was assessed by MTT, cell counting, and EdU. (a, b) PFD decreased the hypoxia-induced HPAAF proliferation assessed by MTT and cell counting (*n* = 5). (c, d) PFD decreased the hypoxia-induced HPAAF proliferation assessed by EdU (*n* = 5). Red: EdU; blue: cell nucleus. Hyp: hypoxia; OD: optical density; PFD: pirfenidone; HPAAFs: human pulmonary arterial adventitial fibroblasts.

As reported previously, human pulmonary arterial adventitial fibroblasts (HPAAFs) display significant proliferation and migration during the pathological remodeling process caused by HPH [3]. HPAAFs were treated with pirfenidone (PFD) at 0.1mg/ml or 0.2mg/ml before hypoxia exposure to assess cellular proliferation by use of the MTT assay, cell counting, and EdU assay. The results demonstrated that compared with the control, hypoxia induced HPAAF proliferation, whereas PFD significantly attenuated cell proliferation (Figure 1(a), *P* < 0.001; Figure 1(b), *P* < 0.001; Figure 1(c), *P* = 0.001).

### 3.2. PFD Inhibited Hypoxia-Induced HPAAF Migration

**Figure 2:**
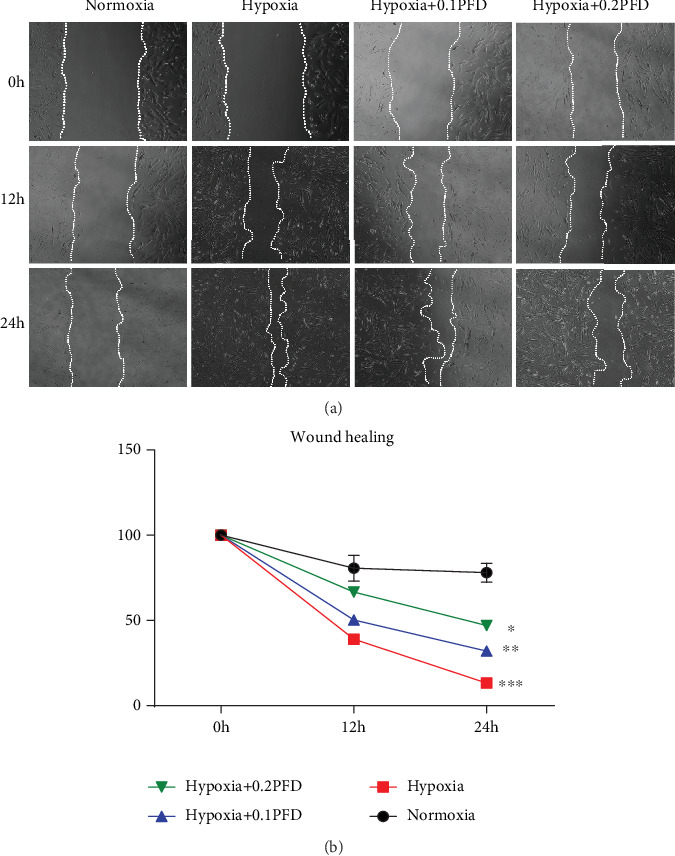
PFD treatment reduced HPAAF migration after 12 h or 24 h hypoxia exposure. HPAAFs were stimulated by PFD before hypoxia, and cell migration was analyzed by wound healing assay. (a, b) Results and quantification of wound healing assay in HPAAFs following 12 h and 24 h of treatment (*n* = 3). ∗*P* < 0.001 vs. normoxia; ∗∗*P* = 0.004 vs. hypoxia; ∗∗∗*P* < 0.001 vs. hypoxia. PFD: pirfenidone; HPAAFs: human pulmonary arterial adventitial fibroblasts.

To evaluate the effects of PFD on HPAAF migration, we employed a wound healing assay.  Figure 2 indicates that hypoxia accelerated fibroblast migration compared with the control (*P* < 0.001). However, PFD treatment reduced fibroblast migration after hypoxia exposure for 12 h or 24 h.

### 3.3. PFD Decreased the Hypoxia-Induced Expression of a-SMA and Procollagen I in HPAAFs

**Figure 3:**
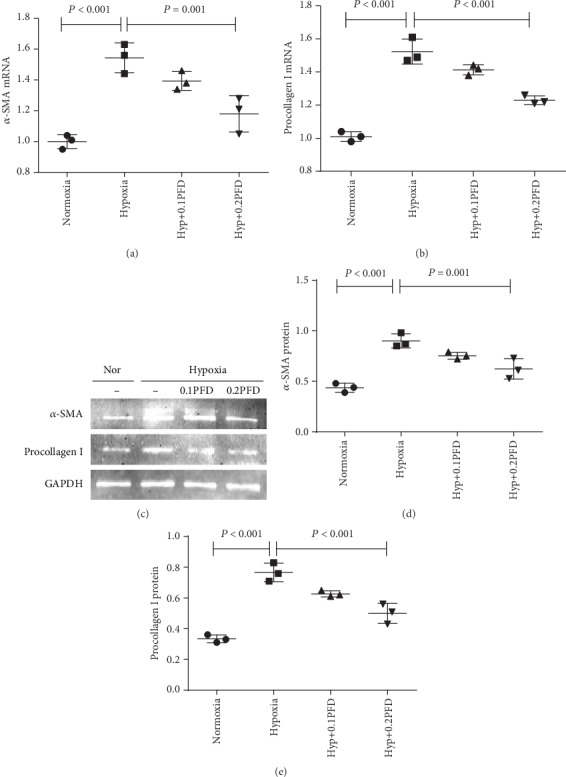
PFD significantly inhibited the mRNA and protein expression of a-SMA and procollagen I (COL1A1). (a, b) The mRNA expression of a-SMA and procollagen I (COL1A1) was assessed by RT-PCR. (c–e) The protein expression of a-SMA and procollagen I (COL1A1) was assessed by western blot analysis. Nor: normoxia; Hyp: hypoxia; PFD: pirfenidone; a-SMA: smooth muscle a-actin.

The transformation of fibroblasts into myofibroblasts leads to the increased expression of contractile proteins, such as *α*-smooth muscle actin (a-SMA) and procollagen I (COL1A1), which are the most frequently utilized myofibroblast markers [22]. Thus, we analyzed the effect of PFD on the expression of a-SMA and procollagen I in HPAAFs. The mRNA and protein expression of a-SMA and procollagen I was significantly increased in the hypoxia group compared with the control group; this upregulation in expression was significantly attenuated by PFD at 0.1mg/ml or 0.2mg/ml (Figure 3, *P* < 0.05).

### 3.4. PFD Inhibited Hypoxia-Induced p38 Phosphorylation

**Figure 4:**
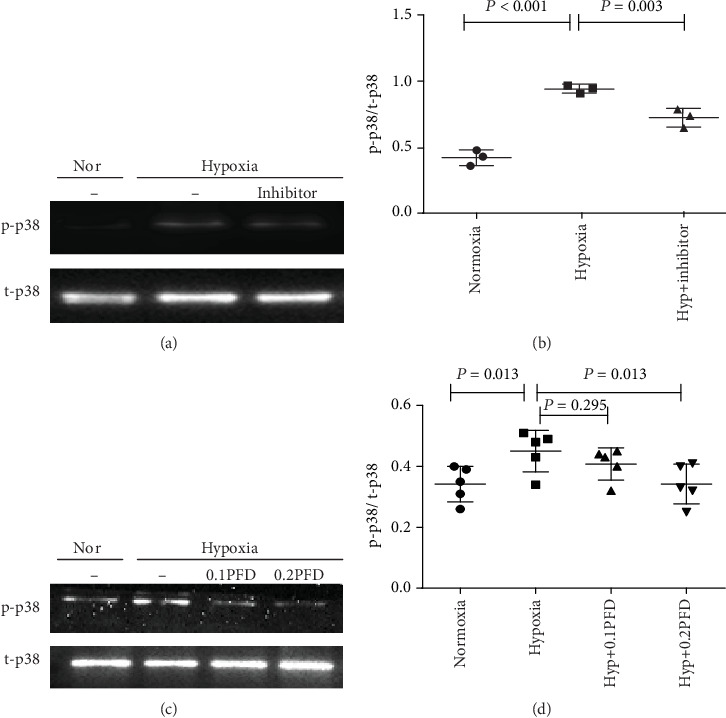
PFD treatment reduced hypoxia-induced p38 phosphorylation in HPAAFs. (a, b) Hypoxia increased the phosphorylation of p38, while preincubation with p38 inhibitor (SB203580, 5 mM) reduced the expression of p-p38 (*n* = 3). (c, d) PFD treatment reduced hypoxia-induced p38 phosphorylation in HPAAFs (*n* = 5). Nor: normoxia; Hyp: hypoxia; SB203580: p38 inhibitor; PFD: pirfenidone; HPAAFs: human pulmonary arterial adventitial fibroblasts.

p38 plays a critical role in HPAAF proliferation under hypoxic conditions [7]. First, we evaluated the phosphorylation of p38 induced by hypoxia by western blot analysis. As shown in Figures 4(a) and 4(b), hypoxia increased the levels of p-p38 in HPAAFs compared with the control group (*P* < 0.001), while SB203580, a p38 inhibitor, significantly attenuated hypoxia-induced phosphorylation of p38 (*P* = 0.003). Similarly, PFD reduced p38 phosphorylation in HPAAFs (Figures 4(c) and 4(d); *P* = 0.013).

### 3.5. PFD Reduced the Hypoxia-Induced Phosphorylation of p38 through the NOX4/ROS Pathway

**Figure 5:**
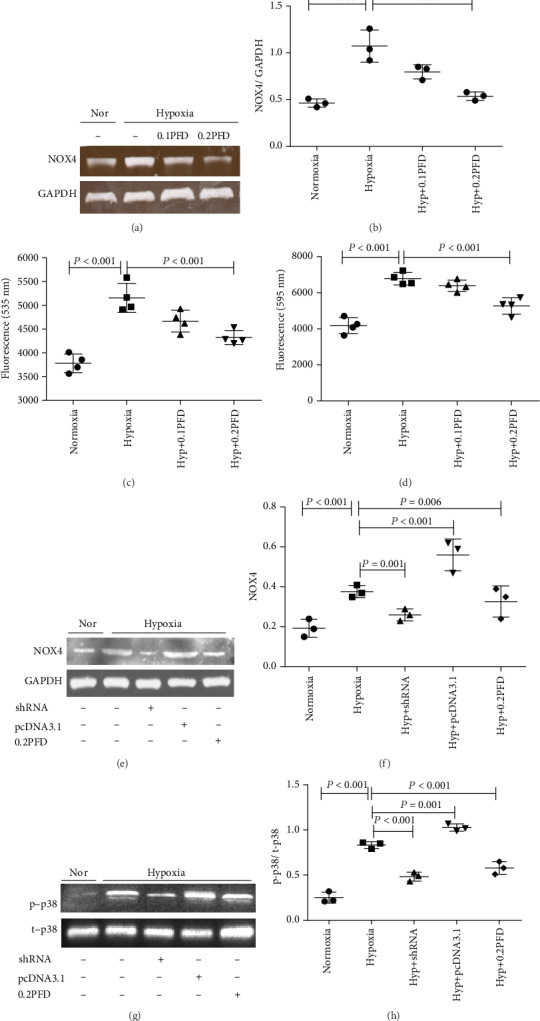
The PFD reduced the phosphorylation of p38 through the NOX4/ROS signaling pathway in HPAAFs exposed hypoxia. (a–d) Compared with normoxia, hypoxia increased the expression of NOX4 and ROS, while PFD reduced NOX4 protein expression and ROS expression in cultured HPAAFs (*n* = 3). (e, f) NOX4 expression was effectively interfered by shRNA or pcDNA3.1, and PFD significantly decreased the hypoxia-induced NOX4 expression (*n* = 3). (g, h) NOX4 inhibition or PFD significantly decreased the phosphorylation of p38 induced by hypoxia in HPAAFs, and overexpression of NOX4 by pcDNA3.1 increased the expression of p-p38 (*n* = 3). Nor: normoxia; Hyp: hypoxia; PFD: pirfenidone; p-p38: phosphorylated p38; t-p38: total p38; HPAAFs: human pulmonary arterial adventitial fibroblasts.

Reactive oxygen species (ROS) are widely associated with vascular remodeling and increased pulmonary arterial pressures [23]. NOX4, the most important isoform of NOX in HPAAFs, has gained attention as a primary source of intracellular ROS [12, 24]. We found that compared with the control, hypoxia significantly increased the expression of NOX4 (*P* < 0.001) and ROS (*P* < 0.001), which were reduced by the administration of PFD (*P* < 0.001) (Figures 5(a)–5(d)). To further clarify the effect of NOX4 on p38 phosphorylation, shRNA and pcDNA3.1 were used to alter levels of NOX4. To verify the efficacy of the knockdown, western blot analysis showed that NOX4 shRNA reduced NOX4 expression (*P* = 0.001); conversely, pcDNA3.1 for NOX4 significantly increased its expression (*P* < 0.001) (Figures 5(e) and 5(f)). Moreover, NOX4 inhibition reduced the phosphorylation of p38 (*P* < 0.001), while overexpression of NOX4 increased the phosphorylation of p38 in HPAAFs (*P* = 0.001) (Figures 5(g) and 5(h)). This hypoxia-induced upregulation of NOX4 (*P* = 0.006) and p-p38 was significantly reduced by PFD administration (*P* < 0.001) (Figures 5(e)–5(h)). These results indicate that PFD downregulates the phosphorylation of p38 through the NOX4/ROS pathway.

### 3.6. The Inhibition of Rac1 by Pirfenidone Reduced the Hypoxia-Induced Upregulation of ROS

**Figure 6:**
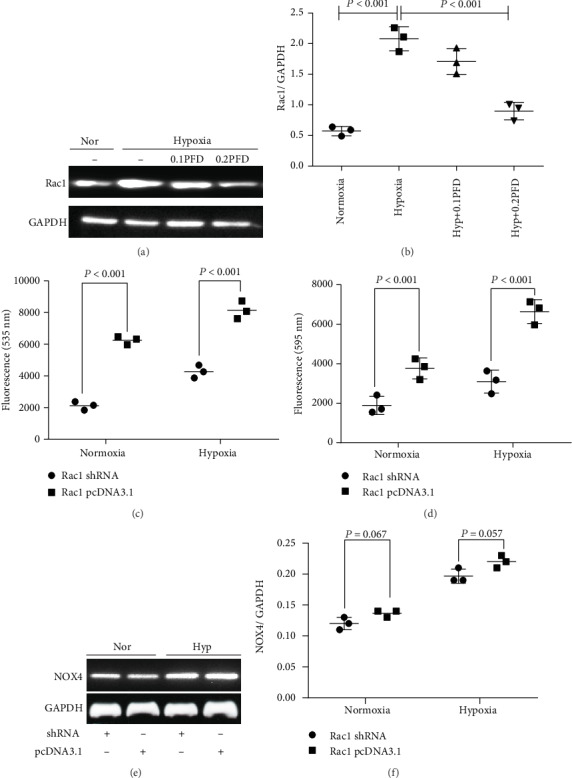
PFD treatment reduced the expression of Rac1, and inhibition of Rac1 reduced ROS without effect on NOX4 in HPAAFs exposed to hypoxia. (a, b) Compared with the control, hypoxia increased Rac1 expression, while PFD treatment reduced the expression of Rac1 in HPAAFs (*n* = 3). (c, d) Inhibition of Rac1 reduced ROS expression and overexpression of Rac1 increased ROS expression (e, f) with no effect on NOX4 expression (*n* = 3). PFD: pirfenidone; Nor: normoxia; Hyp: hypoxia; PFD: pirfenidone; HPAAFs: human pulmonary arterial adventitial fibroblasts.

Hypoxia significantly increased the protein expression of Rac1 (*P* < 0.001), which could be suppressed by PFD administration (*P* < 0.001) (Figures 6(a) and 6(b)). Overexpression of Rac1 obviously increased ROS expression (*P* < 0.001), while inhibition of Rac1 decreased ROS expression (*P* < 0.001) (Figures 6(c) and 6(d)). To confirm the efficacy, specific shRNA and pcDNA3.1 were used to modulate Rac1 levels (Figures 6(e) and 6(f)). Rac1 had no effect on the expression of NOX4 in HPAAFs. These results collectively demonstrated that Rac1 modulates ROS expression, but there was no direct relationship between NOX4 and Rac1.

## 4. Discussion

In the present study, we investigated the role and mechanism of PFD in the proliferation, migration, and fibrosis of HPAAFs. We firstly reported that PFD could prevent the proliferation and migration of HPAAFs and could reduce the expression of *α*-smooth muscle actin (a-SMA) and procollagen I (COL1A1). In addition, we showed that PFD played a critical role in HPH through the NOX4/ROS/p38 signaling pathway. We also found that Rac1 could also decrease hypoxia-induced phosphorylation of p38, without a cross-interaction with NOX4. These data suggest that PFD has therapeutic potential as a drug to treat hypoxia-induced HPH.

To date, the precise mechanism of HPH has not been fully elucidated, and the focus has shifted to the cellular changes within the adventitia. The adventitial stroma is now considered to be the most complex and heterogeneous compartment of the vessel wall, and the most abundant cells within it are PAAFs. An increasing number of studies support the argument that the pulmonary arterial adventitia acts as biological processing centers for the remodeling of HPH. In response to hypoxia, PAAFs are the first cells to be activated, leading to the recruitment of inflammatory cells and the release of key regulators, such as monocyte chemotactic protein-1 (MCP-1/CCL2), interleukin-6 (IL-6), stromal cell-derived factor-1 (SDF-1/CXCL12), and matrix metalloproteinases (MMPs), which are responsible for medial smooth muscle cell (SMC) growth [6]. Hypoxia causes fibroblasts to differentiate into myofibroblasts, which migrate from the adventitia to the media and the intima. This results in increased extracellular matrix (ECM) protein expression, thus contributing to pulmonary arterial remodeling. Furthermore, PAAFs also induce the inflammation of pulmonary vessels and promote the progression of HPH. Adventitial fibroblasts can regulate macrophage activation through paracrine IL-6 to activate signal transducer and activator of transcription 3 (STAT3) and hypoxia-inducible factor 1 (HIF1) signaling in hypoxia-induced HPH [25]. This proliferation and migration of PAAFs in response to hypoxia can be inhibited or even reversed by fluvastatin [26]. Changes of metabolism and inflammation in fibroblasts are the major causes of pulmonary artery remodeling in HPH [27]. Therefore, PAAFs might be a target for the therapeutic intervention of HPH. Our results indicate that PFD suppresses the proliferation and migration of HPAAFs in hypoxia. The adventitial layer of the pulmonary artery thickens because of the hypoxia-induced proliferation of resident PAAFs, while the fibroblasts in systemic artery adventitia do not exhibit a similar response [28].

The p38 mitogen-activated protein kinase (MAPK) pathway plays an essential role in a variety of cellular functions. Upregulated expression of p38 coupled with increased proliferation and migration of PAAF within the pulmonary artery is a critical component of the pathophysiology of pulmonary arterial remodeling in HPH. We found that hypoxia increased the phosphorylation of p38 in HPAAFs, which was significantly attenuated by PFD at 0.2mg/ml. The phosphorylation of p38 induced by hypoxia was mediated by intracellular ROS, which was mostly produced by NOX. Seven NOX isoforms are encoded in the genome, and four isoforms, NOX1, NOX2, NOX4, and NOX5, are expressed in vascular cells [29]. NOX enzymes are considered to be the primary sources of ROS [30]. Evidence supports the view that NOX4 plays a crucial role in many cardiovascular diseases [31], especially in the pathogenesis of HPH [32–34]. In addition to that of mice and rats, NOX4 is localized primarily in the perinuclear space in all three layers of the vascular wall in humans, and NOX4 expression is increased after exposure to hypoxia in human HPH [35]. We also showed that the expression of NOX4 was upregulated in PAAFs under hypoxic conditions, and PFD was able to reduce this upregulation of NOX4, while also decreasing the expression of ROS. Moreover, knockdown of NOX4 with shRNA reduced the phosphorylation of p38, while overexpression of NOX4 with pcDNA3.1 increased the levels of phosphorylated p38.

The Ras-related C3 botulinum toxin substrate (Rac1) is one of the 20 members of the Rho superfamily of small GTPases, which are classified as six subfamilies: Rnd, Rho, Rac, Cdc42, RhoBTB, and RhoT/Miro [36]. Rac may exist as one of four isoforms (Rac1, Rac1b, Rac2, and Rac3); Rac1 is ubiquitously expressed in nonhematopoietic cells and is likely the major isoform involved in NOX activation [37]. Following activation, Rac1 translocates to the membrane of cells and activates the NOX4 complex. Martyn et al. have shown that knockdown or pharmacological inhibition of Rac1 does not affect the constitutive activity of NOX4, which suggests that NOX4 is Rac1-independent in epithelial cells [38]. In contrast, Gorin et al. in 2003 reported that Rac1 regulates the generation of ROS by stimulation of a NOX4-based NADPH oxidase [39] and suggested that NOX4 activation is part of a Rac1-dependent signaling pathway in mesangial cells. Our results suggested that there was no direct cross modulation between NOX4 and Rac1. We speculated that Rac1 was probably a major regulator of the NOX4-Rac1 complex following assembly and activation in HPAAFs. The discrepancies between our results and Gorin's in terms of the relationship between NOX4 and Rac1 could be explained by the differences in cell type, but further studies are needed for confirmation.

To determine the mechanism of PFD in modulating proliferation, migration, and fibrosis in HPAAFs, we used two concentrations of PFD (0.1mg/ml and 0.2mg/ml). The results suggested that PFD exerted its effects in a concentration-dependent manner. A previous study showed that PFD targets the p38 MAPK signaling pathway and inhibits profibrotic gene expression in human dermal myofibroblasts [40]. Moreover, PFD inhibits phenotypic switching and collagen synthesis in a ROS-dependent fashion in human pulmonary artery smooth muscle cells (HPASMCs) in idiopathic pulmonary fibrosis (IPF) [41]. We found that compared with the hypoxic group, PFD reduced the expression of a-SMA and procollagen I (COL1A1), which suggested an antifibrotic role of PFD.

A potential limitation of this study was that it was only performed in vitro. Thus, the next major approach would be to conduct experiments in vivo. In addition, we could not determine the precise relationship between NOX4 and Rac1.

## 5. Conclusions

**Figure 7:**
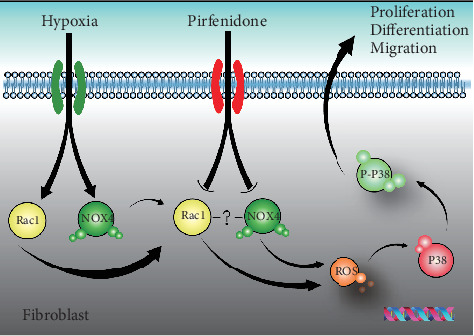
The role of PFD on HPAAFs in hypoxia. In the human pulmonary artery adventitial fibroblasts, PFD reduced hypoxia-induced cell proliferation, migration, and differentiation through the NOX4/ROS/P38 signaling pathway. Meanwhile, there was no direct cross effect between NOX4 and Rac1. PFD: pirfenidone; ROS: reactive oxygen species.

Despite some limitations, we confirm that PFD exerts a protective role in HPH through the NOX4/ROS/p38 signaling pathway in HPAAFs (Figure 7). The experiment supports the effectiveness of PFD in halting the proliferation, differentiation, and migration of HPAAFs, and PFD may have therapeutic potential in the prevention and treatment of HPH.

## Data Availability

The datasets used and analyzed during the current study are available from the corresponding author on reasonable request.
